# Impact of a Mobilized Stress Management Program (Pep-Pal) for Caregivers of Oncology Patients: Mixed-Methods Study

**DOI:** 10.2196/11406

**Published:** 2019-05-03

**Authors:** Alaina L Carr, Jacqueline Jones, Susan Mikulich Gilbertson, Mark L Laudenslager, Jean S Kutner, Kristin Kilbourn, Timothy S Sannes, Benjamin W Brewer, Elissa Kolva, Tanisha Joshi, Nicole Amoyal Pensak

**Affiliations:** 1 Department of Psychology University of Colorado-Denver Denver, CO United States; 2 College of Nursing University of Colorado Anschutz Medical Campus Aurora, CO United States; 3 Department of Psychiatry University of Colorado Anschutz Medical Campus Aurora, CO United States; 4 Division of General Internal Medicine Department of Medicine University of Colorado School of Medicine Aurora, CO United States; 5 Division of Hematology Department of Medicine University of Colorado Anschutz Medical Campus Aurora, CO United States; 6 Division of Medical Oncology Department of Medicine University of Colorado Anschutz Medical Campus Aurora, CO United States; 7 Jersey Shore University Medical Center Hackensack Meridian Health Neptune, NJ United States; 8 Georgetown University Medical Center Washington, DC United States

**Keywords:** advanced cancer caregivers, psychoeducation, mHealth, cancer, bone marrow transplantation, qualitative research, internet, randomized controlled trial, caregivers, neoplasms, telemedicine, clinical trial, phase I

## Abstract

**Background:**

Caregivers of patients with advanced diseases are known to have high levels of distress, including depression and anxiety. Recent research has focused on recognizing caregivers in need of psychosocial support to help them manage their distress. Evidenced-based technological interventions have the potential to aid caregivers in managing distress.

**Objective:**

The objective of our study was to describe caregiver perceptions of the usability and acceptability, and their suggestions for future adaptations, of a mobilized psychoeducation and skills-based intervention.

**Methods:**

This study was a part of a larger trial of a mobilized psychoeducation and skills-based intervention (Psychoeducation and Skills-Based Mobilized Intervention [Pep-Pal]) for caregivers of patients with advanced illness. This substudy used a mixed-methods analysis of quantitative data from all 26 intervention participants and qualitative data from 14 intervention caregivers who completed the Pep-Pal intervention. The qualitative semistructured individual interviews, which we conducted within the first 4 weeks after participants completed the intervention, assessed the acceptability and usability of Pep-Pal. Additionally, the qualitative interviews provided contextual evidence of how the intervention was helpful to interviewees in unanticipated ways. We conducted applied thematic analysis via independent review of transcripts to extract salient themes.

**Results:**

Overall, caregivers of patients with advanced cancer deemed Pep-Pal to be acceptable in all Web-based sessions except for Improving Intimacy. Caregivers perceived the program to be of use across the areas they needed and in others that they had not anticipated. Caregiver recommendations of key changes for the program were to include more variety in caregiver actors in sessions, change the title of Improving Intimacy to Improving Relationships, provide an audio-only option in addition to video, and change the format of the mobilized website program to a stand-alone mobile app.

**Conclusions:**

The valuable feedback in key areas from individual interviews will be integrated into the final version of Pep-Pal that will be tested in a fully powered randomized clinical trial.

**Trial Registration:**

ClinicalTrials.gov NCT03002896; https://clinicaltrials.gov/ct2/show/NCT03002896 (Archived by WebCite at http://www.webcitation.org/76eThwaei)

## Introduction

### Background

There are over 40 million caregivers in the United States [1], and this number will only increase over time [2]. Caregivers provide uncompensated support for loved ones at a value of over US $450 billion per year [2] and lost income equivalent to over US $300,000 per lifetime [3]. Over half of caregivers report feeling overwhelmed by their responsibilities [2,4]. Caregivers have been termed “silent patients,” neglecting to seek treatment for themselves while taking care of their loved ones. For this study, we defined primary caregiver as the person in the patient’s life who was primarily responsible for care decisions, was emotionally invested in the patient’s care, and provided instrumental care, such as transportation. Caregivers of patients receiving hematopoietic stem cell transplant (HSCT), of patients enrolled in phase 1 oncology clinical trials, and of patients with advanced cancer experience significant distress [5-7]. Besides the transplant process, patients who undergo HSCT commonly have sexual dysfunction [8-10], which can also contribute to caregiver distress.

Caregivers have been found to be reluctant to participate in in-person support services because of the extra burden of time constraints [11]. There are barriers to accessing treatment, and consequently there is strong support for developing novel and *convenient* behavioral health interventions to support caregivers in coping with caretaking responsibilities and reducing depression and anxiety [5]. Use of technology to deliver innovative and convenient behavioral health interventions to support cancer caregivers can improve coping and reduce depression and anxiety without the added burden of having to attend a face-to-face session [5,12]. According to the US National Alliance for Caregiving, a large majority of family caregivers believed that using technologies such as video phone systems and a caregiving coordination system would be personally beneficial, save them time, make caregiving easier logistically, increase self-efficacy, and reduce stress [13]. In particular, mobile technologies (eg, telehealth) have been effectively implemented in family caregiver populations without face-to-face interactions and may help to overcome some logistical and geographical barriers to obtaining support [14,15].

Telehealth is a mode of delivering health care services through telecommunication and is commonly used to deliver educational interventions, consultation services, and behavioral interventions [16]. It can be used as a means of improving social support, collecting care management data, monitoring symptoms, and delivering clinical care [15]. In a review assessing telehealth tools and support to family caregivers, more than 95% of the 65 studies reported significant improvement in psychosocial outcomes [15]. Telehealth studies involving rural family caregivers, as well as telehealth studies conducted in a home setting, found significant improvements in psychological health and quality of life of family caregivers. Additionally, family caregivers reported high levels of satisfaction and comfort with using telehealth [14,15]. These findings suggest that family caregivers who provide around-the-clock care and symptom monitoring can use telehealth interventions for efficient care while reducing the burden of traveling to medical clinics.

While evidence on the effects of telehealth interventions on family caregivers is encouraging, further attention is needed to identify the most effective technologies for family caregivers of cancer patients. Furthermore, because rates of mobile phone use are high among socioeconomically disadvantaged populations [17], mobile technologies present an optimal intervention strategy for targeting caregivers with financial limitations and other barriers to accessing in-person care. As technologies continue to emerge, engaging caregivers still remains a significant challenge [18-21]. To our knowledge, there are no evidence-based interventions to help caregivers manage their distress using technological platforms that can be disseminated widely.

### Evidence-Based Intervention

Recent studies have shown that brief interventions can be effective in reducing distress among caregivers of allogenic HSCT (allo-HSCT) patients [22]. Allo-HSCT patient have certain cancers of the blood or bone marrow and receive an infusion of a human leukocyte antigen-matched donor stem cell. Providing strategies to improve communication with their loved ones and intimacy after transplant may help caregivers better adjust to relationship changes. To advance knowledge in this area and overcome limitations of available caregiver resources, we completed a randomized controlled trial (RCT) of an in-person skills-based intervention with caregivers of allo-HSCT patients [5]. The brief intervention, Psychoeducation, Paced Respiration and Relaxation (PEPRR), was shown to reduce perceived stress in caregivers (primary outcome) with reductions in depression and anxiety as secondary outcomes [5]. We adapted PEPRR and enhanced it for a mobile-based platform (Psychoeducation and Skills-Based Mobilized Intervention [Pep-Pal]). Based on focus groups and feedback in our preliminary formative mobile health evaluation work, we found that Pep-Pal was feasible and usable among caregivers of patients receiving autologous HSCT (auto-HSCT) [6]. This substudy built upon the formative feasibility and usability study and tested the mobilized intervention, Pep-Pal, in a pilot RCT with caregivers of auto-HSCT patients, caregivers of patients enrolled in phase 1 oncology trials, and caregivers of patients with advanced cancer.

### Objective

The purpose of this study was to continue to establish Pep-Pal as an evidence-based intervention for reducing distress in caregivers of patients with advanced illness by further assessing acceptability and usability of Pep-Pal through qualitative interviews and self-report assessments. The aims of this study were to assess acceptability of Pep-Pal by caregivers based on mean self-reported helpfulness scores, and usability based on the majority of caregivers’ ratings as above average on the usability questionnaire. We evaluated acceptability and usability of Pep-Pal through semistructured qualitative interviews. In addition, we explored ways to improve Pep-Pal based on caregiver feedback via postintervention questionnaires administered to all intervention participants and through qualitative interviews. Feedback about improvements to Pep-Pal will be integrated into a final version to be tested in a fully powered RCT.

## Methods

### Setting

This study was conducted at the University of Colorado Comprehensive Cancer Center, Aurora, CO, USA, a large urban academic medical center with a diverse range of patients with socioeconomic statuses seen from across the state.

### Participants

Participants were eligible to enroll if they identified as a primary caregiver of a patient who was either receiving an HSCT, enrolled in a phase 1 oncology clinical trial, or with a diagnosis of advanced cancer (stage IV, solid tumor). For this study, we defined primary caregiver as the person in the patient’s life who was primarily responsible for care decisions, was emotionally invested in the patient’s care, and provided instrumental care such as transportation. Additional inclusion criteria for participants were (1) age over 18 years, (2) ability to read and speak English, (3) absence of cognitive or psychiatric conditions prohibiting participation (eg, significant developmental or intellectual disability), (4) endorsement of a moderate level of anxiety (eg, ≥8 on the Hospital Anxiety and Depression Scale subscale for Anxiety [HADS-A] [23,24]), and (5) access to a computer, laptop, smartphone, or tablet with internet access. We based the rationale for the screening cutoff score of 8 or above on the HADS-A on clinically significant anxiety symptoms in medical populations [23,24]. There were no other inclusion or exclusion criteria.

### Procedure

We recruited participants over an 11-month period in the HSCT clinic, the Phase 1 Oncology Trials Clinic, and the gastrointestinal, lung, glioblastoma, and genitourinary medical oncology clinics in the study setting. We obtained informed consent alongside a treatment visit or provider appointment. We deemed potential participants to be eligible if they endorsed a total score of 8 or above (moderate level of anxiety) on the HADS-A. Study staff reviewed study procedures, the consent form, and data collection procedures with eligible participants. After participants provided consent, we administered baseline questionnaires. Randomization by permuted block design, set by the study statistician (SMG), was completed after baseline assessment. Participants were randomly assigned to receive either Pep-Pal in addition to treatment as usual or treatment as usual only. Treatment as usual was any support or resources caregivers sought out themselves. Study staff provided access to Pep-Pal (passcode) through email. Caregivers were instructed to watch each session at least once, watch 1 to 2 new sessions per week, and practice skills between sessions. Participants were informed that they could go back and watch sessions as many times as they liked. Study participants filled out postassessment questionnaires delivered via an automated REDCap (REDCap Consortium) email at 12 weeks after enrollment. After postassessment completion, we contacted a subgroup of participants by purposeful selection of 14 intervention completers to conduct a semistructured qualitative interview. This study examined responses to semistructured interviews conducted with 14 intervention completers within 4 weeks after they had completed the Pep-Pal intervention. The trial was approved by the Colorado Multiple Institutional Review Board and registered with ClinicalTrials.gov (NCT03002896).

### Pep-Pal Intervention

Pep-Pal was delivered via a mobilized website that was conveniently accessible anytime by smartphone, computer, tablet, or laptop. Pep-Pal consisted of 9 full-length sessions that were each less than 20 minutes. The 9 sessions were (1) Introduction to Stress Management, (2) Stress and the Mind-Body Connection, (3) How Our Thoughts Can Lead to Stress, (4) Coping With Stress, (5) Strategies for Maintaining Energy and Stamina, (6) Coping With Uncertainty, (7) Managing Relationships, (8) Getting the Support You Need, and (9) Improving Intimacy (Multimedia Appendix 1). Additionally, the website included “Mini-Peps,” brief (<3 minutes each) video guided activities including relaxation exercise modules (eg, body scan, deep breathing, and mindfulness meditation), mood exercises (eg, gratitude exercises), and relationship enhancement activities (eg, communication exercises) (Multimedia Appendix 2).

### Measures

#### Demographic Questionnaire

Each participant completed a demographic questionnaire at baseline that requested information on age, sex, race, ethnicity, marital status, religion, relation to patient, education level, living context (eg, number of children in the household and their ages), duration of caregiving specific to this illness, and patient’s diagnosis.

#### Pep-Pal Usability Questionnaire

The Pep-Pal Usability Questionnaire delivered at postassessment posed 9 questions regarding the experience of using Pep-Pal on a 5-point Likert scale. Higher total scores indicated greater usability (Cronbach alpha=.88).

#### Helpfulness of Intervention Sessions Questionnaire

The Helpfulness of Intervention Sessions Questionnaire, delivered at postassessment, asked 10 questions regarding the helpfulness of each intervention session on a 10-point Likert scale. Higher total scores indicate greater helpfulness (Cronbach alpha=.96).

#### Semistructured Interview

We used a semistructured interview guide (Multimedia Appendix 3) to conduct qualitative interviews.

### Data Analysis

This mixed-methods substudy included analyses of both quantitative and qualitative data. We conducted descriptive statistics on 14 intervention completers’ baseline demographic questionnaires using IBM SPSS version 24 (IBM Corporation). We assessed the usability and acceptability of Pep-Pal using descriptive data that reported means and proportions. We analyzed the qualitative data from interviews, which were audiorecorded and transcribed, using an inductive approach to thematic analysis to draw out broad themes and subthemes within the data [25]. Data analysis involved systematic organization of data through open coding in ATLAS.ti version 8.2.1 (ATLAS.ti Scientific Software Development GmbH). Data analysis also involved repeated continuous comparisons across coded data to identify salient themes. We used a team approach to synthesize and contextualize the data. Team members (ALC, NAP, and JJ) independently reviewed the transcripts and met biweekly to discuss emerging themes, discrepancies, and alternative explanations. Ongoing modification of the conceptual framework of themes was a fundamental part of the analytic process. Informational saturation was reached when no new themes emerged regarding key outcomes [26].

## Results

### Participant Characteristics

**Figure 1:**
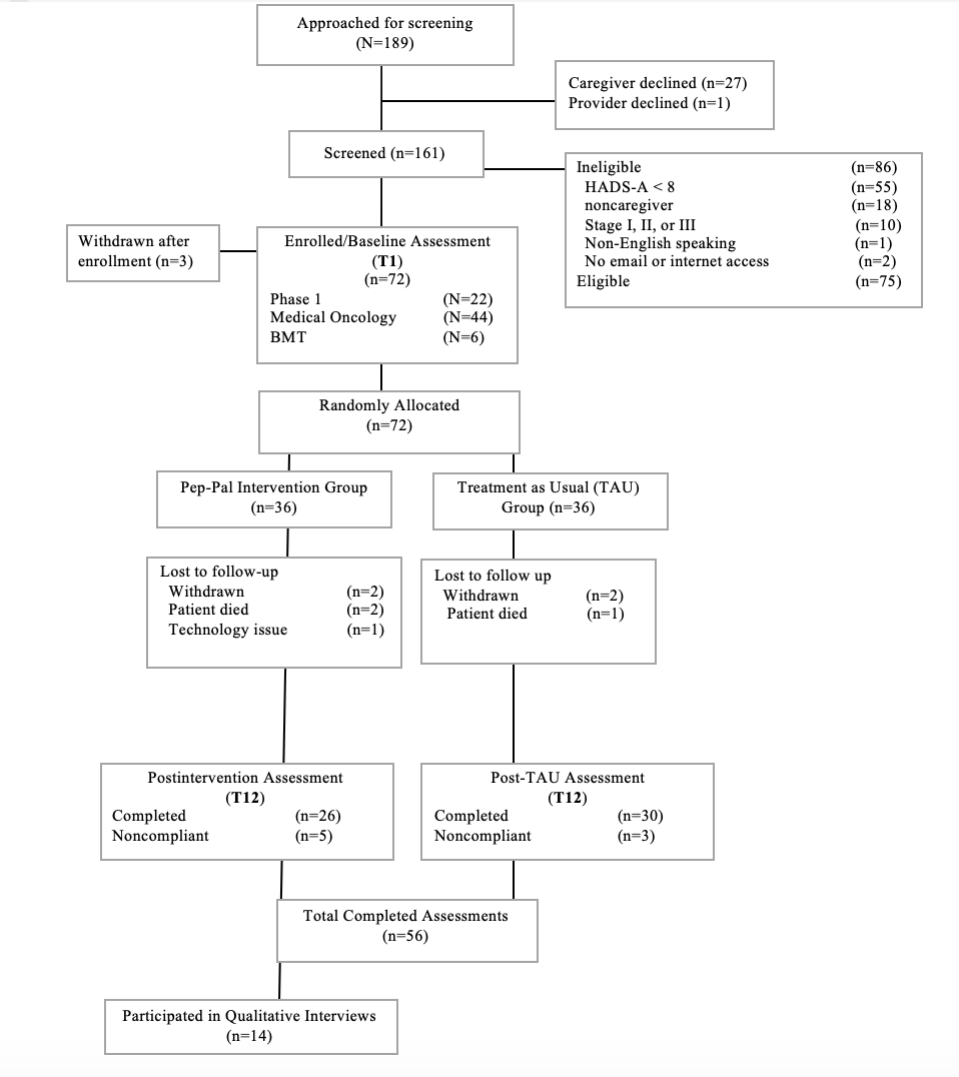
Study flow. BMT: bone marrow transplantation; HADS-A: Hospital Anxiety and Depression Scale subscale for Anxiety.

We approached 189 caregivers for study screening across all clinics. A total of 56 caregivers were enrolled and completed assessments, of whom 14 participated in semistructured interviews. All participants were recruited through medical clinics or referred by their medical team. Figure 1 shows the flow of participants through the study.

Table 1 lists demographic characteristics. Characteristics of caregivers who participated in semistructured interviews were representative of characteristics of those in the larger trial and were not statistically significantly different from the remainder of participants in the trial regarding age, education, relationship status, and race/ethnicity. Most participants were female, at least college educated, married, employed full-time or part-time, and white.

### Acceptability of Pep-Pal

**Figure 2:**
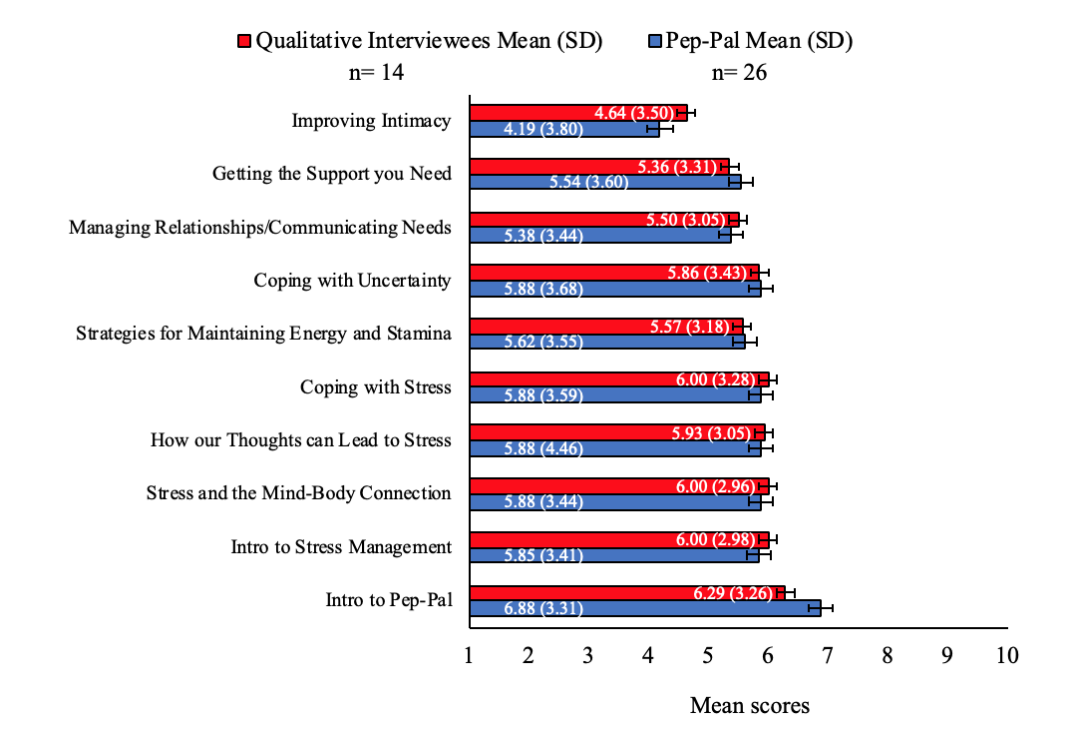
Mean scores on the Helpfulness of Intervention Sessions Questionnaire by group. A score of 10 indicates a “very helpful” session and 1 indicates a “not at all helpful” session. Error bars are standard deviation.

We determined acceptability of Pep-Pal using the Helpfulness of Intervention Sessions Questionnaire and semistructured exit interviews with 14 completers. Participants rated intervention sessions as acceptable as measured by mean helpfulness scores at or above a rating of 5 out of 10 (1=not at all helpful, 5=neutral, 10=very helpful) for all intervention sessions except for the Improving Intimacy session (mean 4.19, SD 3.80; see Figure 2). A qualitative analysis of the interviews indicated that acceptability of the Improving Intimacy session was less about the video content but more about the topic itself, and other participants alluded to intimacy not being a priority when the partner is terminally ill.

When asked about an appropriate session length, 64% (9/14) of the qualitative participants indicated that they were satisfied with the 10- to 20-minute session length, while 28% (4/14) of qualitative participants indicated that full sessions could be 10 minutes or less. Participants were satisfied with the delivery method of Pep-Pal. Despite support for the delivery method of the intervention, 21% (3/14) of caregivers indicated that they would have preferred a more accessible mobile app instead of a Web-based format. These caregivers reported a preference for a mobile app format instead of a Web-based format due to internet connectivity issues during their commute to work.

### Usability of Pep-Pal

**Figure 3:**
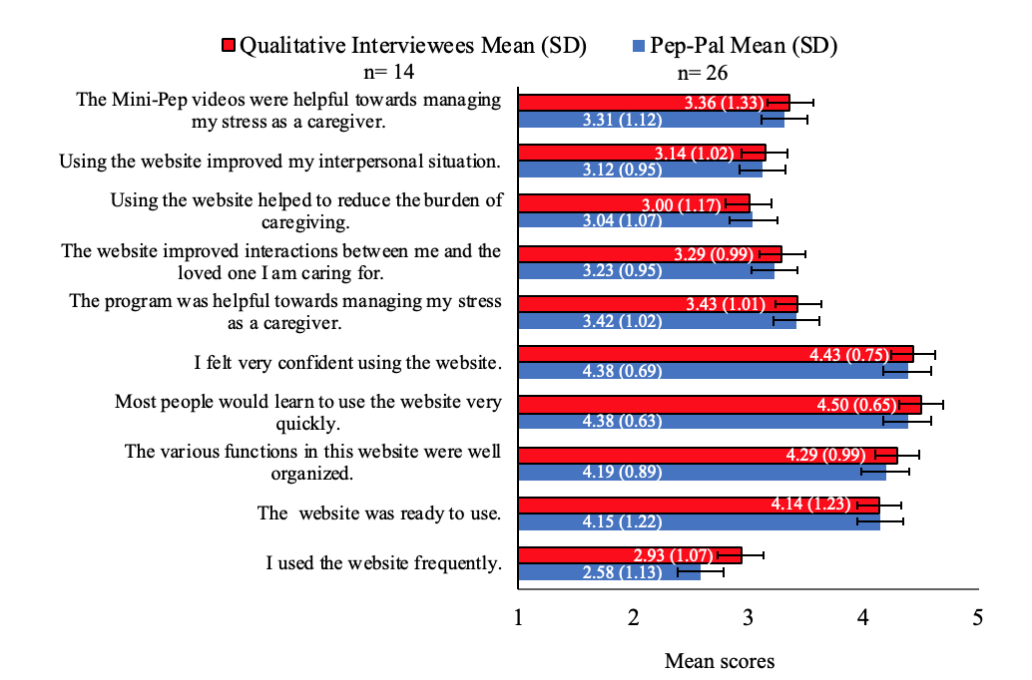
Mean scores on the Pep-Pal Usability Questionnaire by group. A score of 5 indicates “very strongly agree” and 1 indicates “very strongly disagree”. Error bars are standard deviation.

Participants overall felt that Pep-Pal was well organized and easy to navigate (see Figure 3). In terms of the modality used to access Pep-Pal, 64% (9/14) used a computer or laptop, 42% (6/14) used an iPad or tablet, and 21% (3/14) used their smartphone. Several participants reported that they used more than one modality to access Pep-Pal (eg, computer, laptop, tablet, smartphone).

### Thematic Analyses Results

A total of 4 major themes emerged in regard to usability of Pep-Pal for issues related to the caregiver experience: (1) putting the caregiver first, (2) guilt, (3) isolation and loneliness, and (4) latent traumatizing effects. Table 2 shows narrative examples that highlight exit interviewee language, context, and interpretation of usability.

### Putting the Caregiver First

The overarching perspective described was that Pep-Pal was helpful in shifting caregiver focus toward putting the caregiver first. During the program, caregivers described how Pep-Pal helped them shift their focus and remind themselves to prioritize their own mental, physical, and emotional needs. One caregiver described this as “I count as somebody that I need to take care of.” Additionally, caregivers described that the program helped them to balance caregiving with their other daily roles (eg, mother, spouse, friend). Some caregivers indicated that Pep-Pal was helpful in prioritizing time for a spousal or partner role in their relationship with the patient.

### Guilt

The second theme that emerged was that working caregivers felt guilty in falling short of their obligations (eg, because of needing to take time off work). Caregivers described this sense of guilt when taking time off to care for their loved ones or needing to ask coworkers for help. Guilt was also evident when caregivers had to renegotiate caregiving time with family time. Much of the reported caregiver guilt was self-induced and was an internal perception of not living up to their own standards of how they should behave. One caregiver indicated that Pep-Pal helped reframe this sense of guilt by identifying with the term *caregiver* as a way to validate the need to attend the patient’s hospital visits instead of going to work.

**Table 1:** Demographics and key characteristics of caregivers at baseline by group.

Characteristics		Postintervention assessment participants (n=26)	Qualitative interviewees (n=14)
Caregiver age (years), mean (SD)		53.3 (17.7)	52.5 (17.9)
**Patient disease category, n (%)**			
	Enrollment in phase 1 trial	7 (26)	5 (35)
	Lung	14 (53)	5 (35)
	Genitourinary	1 (3)	1 (7)
	Gastrointestinal	1 (3)	1 (7)
	Bone marrow transplantation	3 (11)	2 (14)
	Glioblastoma	N/A^a^	N/A
Female caregiver, n (%)		19 (73)	10 (71)
Married or in a civil union, n (%)		20 (76)	9 (64)
Spouse or civil partner or patient, n (%)		20 (76)	10 (71)
College degree or higher, n (%)		16 (61)	10 (71)
Total annual income ≥US $75,000, n (%)		18 (69)	8 (57)
Living with the patient, n (%)		22 (84)	11 (78)
No. of dependent children, n (%)		17 (30)	6 (42)
**Employment status as a caregiver, n (%)**			
	Full-time	12 (46)	7 (50)
	Part-time	6 (23)	4 (28)
	On leave	N/A	N/A
	Unemployed	2 (7)	1 (7)
	Retired	6 (23)	2 (14)
Patient felt ill prior to diagnosis, n (%)		17 (65)	9 (64)
Chronic health issues prior to diagnosis, n (%)		7 (26)	3 (21)
**Caregiving responsibilities began, n (%)**			
	When patient became ill	20 (35)	5 (35)
	When patient was diagnosed	28 (50)	7 (50)
	Before patient was diagnosed	3 (5)	1 (7)
	Other	5 (8)	1 (7)

^a^N/A: not applicable.

**Table 2:** Summary of qualitative interview results on the usability of Pep-Pal.

Theme and participant ID		Participant type	Quotation
**Putting the caregiver first**			
	1064	Working caregiver, caregiver with a family	She would say, “Stop and write down some things that you think you could do”....I didn’t have time to do that but I did like listening to them and having that time to reflect kind of on my own needs and...mental health.
	1065	Working caregiver, caregiver with a family	Mostly keeping in mind I count as somebody that I need to take care of.
	1071	New caregiver	It’s talking about you need to get out and do things for yourself those things were great reminders.
	1026	Long-term caregiver	It’s very, very difficult to figure out how to basically getting any of my needs met....It’s really difficult because I feel like all of his energy is directed towards fighting his cancer.
**Guilt**			
	1060	Working caregiver, caregiver with a family	I feel guilty you know. And I think to myself, “Man, all of my paid time off has been sucked up from when my husband was in the hospital.”
	1065	Working caregiver, caregiver with a family	If I had to take work off, that’s...really difficult....I could say, “Well, I’m my brother’s primary caregiver so I need to do this” I could feel okay with that. It lessened the guilt.
	1009	Working caregiver	I am working full-time, so I guess so there is a little bit of guilt with that.
**Isolation and loneliness**			
	1075	Working caregiver	During the beginning of my wife’s care it felt very lonely and very isolated and you feel like no one understands.
	1021	Long-term caregiver	The thing that probably most affected me, and still...is the isolation the disease causes.
	1065	Working caregiver, caregiver with a family	That feeling that you’re not alone...that there are people who are dealing with similar things and then if somebody else is dealing...
	1034	Caregiver with a family, new caregiver	It’s just not you...everyone is having some...situation going on and...you’re not alone.
	1060	Working caregiver, caregiver with a family	I just felt really alone in that whole process.
	1026	Long-term caregiver	I just feel sort of lonely in terms of him because he’s not there for me in a way he used to be.
**Latent traumatizing effects**			
	1060	Working caregiver, caregiver with a family	When he first got diagnosed I thought my life was ending....There were emotions at the very beginning...very overwhelming and maybe if I had known about this then, it would have been more helpful for me then.
	1071	New caregiver	It [Pep-Pal] helped with feelings of a little bit of panic every time you get really bad news [laughter]. Like, “Uh-oh” but helped calm me down.
	1075	Working caregiver	The illness in general...you get new information that might not be positive. And then trying to reframe it....“Okay, this is the new normal” and many times when something challenges the new normal...and you have to reset.
	1026	Long-term caregiver	It’s not...easy...to deal with initially....You see people walking around traumatized....Initially, you think, oh, we’ll just do this...and then we’ll go back to our life....But going from shock to...start caregiving immediately.

#### Isolation and Loneliness

The third theme emerging was a negative sense of isolation and loneliness from taking on the primary caregiver role. Most caregivers reported feelings of social isolation and feeling that “no one understands the emotional and physical demands in the progress of being a caregiver.” One caregiver alluded to her “loneliness” as related to the changes in her relationship with her husband and how he could no longer fulfill a supportive role given his disease prognosis. Caregivers indicated that Pep-Pal was helpful in normalizing many isolating aspects of the caregiving experience, such as the unpredictability of daily caregiving responsibilities. Caregivers also described Pep-Pal as being helpful in providing a sense of social cohesion with other caregivers’ experiences, notably without connecting them to other caregivers. Many caregivers expressed a desire for a chatroom feature within Pep-Pal as an additional means of social support.

#### Latent Traumatizing Effects

The fourth theme emerging from caregivers’ comments was a sense of latent trauma or assault with the patient’s terminal illness trajectory. ALC and JJ extracted the lay terms “assault,” “trauma,” and “shock” from caregiver qualitative interviews to contextualize the theme of latent traumatizing effects. Caregivers characterized their caring for a loved one with advanced cancer as heightened arousal, as negative affectivity and mood, and as a trauma itself. This was greater in caregivers’ descriptions of feelings of trauma upon initially hearing about their loved one’s terminal diagnosis. Anticipatory grief, defined as reduced levels of preparedness for their loved one’s imminent death [27], was reported in caring for a loved one with a terminal disease and adjusting to “the new normal” of their daily routines that involved frequent medical visits. Overall, most caregivers described “making sense” of the latent traumatizing effects of caring for their loved one as the biggest mental, physical, and emotional challenge in caregiving.

### Caregiver-Recommended Future Adaptations for Pep-Pal

Three main suggestions emerged (Multimedia Appendix 4). The first suggestion, to change the name of the session, resulted from a mixed response to the full-length Improve Intimacy session. Some interviewees (3/14, 21%) indicated that the intimacy session was not as helpful or relevant to their situation due to patient prognosis or identifying with a nonspousal role with their patient. Alternatively, several interviewees (4/14, 28%) indicated that the intimacy session provided a new perspective on redefining intimacy to include nonsexual activities to recapture meaning in their relationship.

The second suggestion to improve Pep-Pal was to include *different actors* to represent various caregiver demographics. One male caregiver recommended including different sex caregivers in Pep-Pal videos to better tailor the caregiver experiences. Despite the desire to have multiple caregivers featured in Pep-Pal videos, interviewees felt that the “caregiver” featured in the videos normalized and validated *isolating and lonely elements* of the caregiving experience. For example, one caregiver expressed frustration around meal planning with her loved one:

It’s just these are common things that happen...I didn’t know that anybody else has had that very same thing where you’ll go “here’s your dinner” [laughter] or just a wide variety of things...You know you can’t take care of your own health needs sometimes because you can’t get out.

The third suggestion to improve Pep-Pal was contingent on the full-time employment of caregivers. Employed caregivers indicated they would have preferred a mobile app with audio features as an additional way to navigate through the videos on their commute to work. These interviewees were also the only exit interviewees to use their smartphone as their sole modality in viewing the program sessions. Caregivers recommended including an audio component as a means to further integrate the skills from Pep-Pal into their full schedules.

## Discussion

### Principal Findings

Results from quantitative data and individual interviews supported the acceptability and usability of Pep-Pal.

This caregiver feedback highlights the ease of use of the Web-based platform modality and convenience that prior literature recommended for evidenced-based intervention platforms [5,28,29]. Within the specific areas of improving stress management, improving relationships, and the use of the Mini-Peps, caregivers rated the usability of Pep-Pal as “neutral.” Despite neutral ratings, other contextual evidence supports the notion that, overall, the study was positive. For example, user engagement in various sessions related to stress and to getting support, and at least one Mini-Pep, provided more contextual evidence of how the intervention was helpful. In particular, qualitative interviews addressing how the intervention was helpful emphasized how interviewees found Pep-Pal to be helpful in unanticipated ways. The themes of putting the caregiver first, guilt, isolation and loneliness, and latent traumatizing effects of caregiving indicate how Pep-Pal helped participants reconceptualize elements of self-care and acknowledge guilt as a stressor, which is overlooked in this population. The fourth major salient theme, latent traumatizing effects, has been reflected in prior literature as knowledge of a loved one’s advanced cancer diagnosis, and their prognosis is perceived as a traumatic event that can result in anticipatory grief [30]. These overarching themes further emphasize the multidimensional supportive needs of family caregivers and support the usability of Pep-Pal as helpful in addressing psychological, social, mental, and emotional supportive needs for caregivers.

We will integrate feedback from individual interviews into the final version of Pep-Pal to further enhance the helpfulness of the program for caregivers. Based on these interviews, it will be important to include session content or resources on grief to help caregivers process their loved one’s illness and prognosis. For working caregivers and caregivers with families, a full-length session on communication about their loved one’s illness to children and coworkers would be helpful in framing difficult discussions. Many caregivers reported feelings of isolation and loneliness in their caregiving role and felt that Pep-Pal was helpful in normalizing these elements of the caregiver experience. An additional feature of the program such as online chatrooms for caregivers to seek social support from one another may help to further mitigate these feelings of loneliness. Lastly, working caregivers expressed a desire for a mobile app of the program in addition to audio sessions, which would enhance the convenience of Pep-Pal. Variations in types of caregivers featured in sessions (eg, male and female) would also further tailor Pep-Pal to fit individual user needs.

We used a mixed-methods approach to further assess intervention participants’ reasons for their below-average ratings of the Improving Intimacy session. Feedback was less suggestive of improving the video content itself and more indicative of how variable the topic of intimacy is within the types of caregiver-patient relationships. Several interviewees indicated that the intimacy session provided a new perspective on how intimacy can be redefined to include nonsexual activities to recapture meaning in their relationship, which was the main goal of the Improving Intimacy session. The session was not exclusively tailored to the physical act of intimacy but broadly discussed having caregivers redefine intimacy (eg, holding hands, cooking dinner together, or taking a long walk together) in their own relationship (regardless of whether the patient is their significant other, or their child or parent, for example). As a result, we will change the title of the session to Improving Relationships in the final version.

It is important to note that, despite positive perceptions of helpfulness in the program, this program is one of many forms of care and is not a “one-size-fits-all” model. Pep-Pal is geared toward caregivers who cannot physically attend in-person support or have limited time to get to the care they need. This program is one modality in addressing how evidenced-based strategies can be disseminated in a convenient, cost-effective platform.

### Limitations

The study had several limitations. First, most of the intervention caregivers were white, female, spousal caregivers, which might limit the generalizability of the results. Second, this study involved a small qualitative sample of bone marrow transplantation intervention caregivers, which might neglect to highlight the experiences of this type of advanced cancer caregiver in Pep-Pal. Third, technological interventions can yield their own disadvantages. For example, working caregivers described internet connectivity issues when using the Web-based platform on their commute to work.

### Conclusion and Future Directions

We will integrate suggestions for improvement based on the results of this study into the final version of Pep-Pal. Specifically, on the basis of qualitative caregiver feedback, we will add a chatroom feature, audio sessions, content on grief, diversity in caregiver actors, and communication strategies. In addition, we will change the title of the Improving Intimacy session to Improving Relationships. The next step is to demonstrate the efficacy of a mobile app version of Pep-Pal in a fully powered RCT with advanced cancer caregivers. Ultimately, the goal will be to conduct a larger, multisite effectiveness implementation study of Pep-Pal.

## Appendix

Multimedia Appendix 1 Screenshot of some of the full-length Pep-Pal sessions.

Multimedia Appendix 2 Screenshot of some of the Mini-Pep sessions.

Multimedia Appendix 3 Semistructured interview guide.

Multimedia Appendix 4 Summary of caregiver-recommended future implementations for Pep-Pal.

## Abbreviations

allo-HSCT: allogenic hematopoietic stem cell transplant
auto-HSCT: autologous hematopoietic stem cell transplant
HADS-A: Hospital Anxiety and Depression Scale subscale for Anxiety
HSCT: hematopoietic stem cell transplant
Pep-Pal: Psychoeducation and Skills-Based Mobilized Intervention
PEPRR: Psychoeducation, Paced Respiration and Relaxation
RCT: randomized controlled trial
