# Supplementary figures and images for: Impact of a Mobilized Stress Management Program (Pep-Pal) for Caregivers of Oncology Patients: Mixed-Methods Study
Source: JMIR Cancer. 2019 May 3;5(1):e11406. doi: 10.2196/11406 (PMC6524452; doi:10.2196/11406)

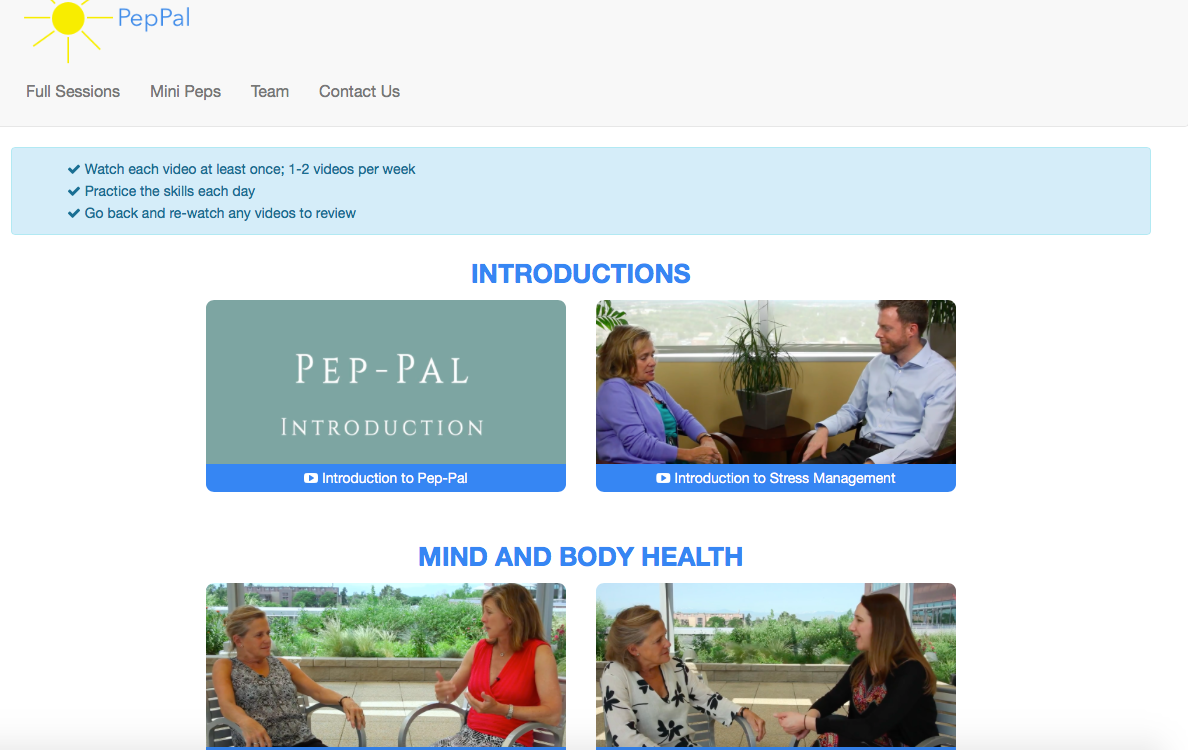

Supplement: Multimedia Appendix 1 [file cancer_v5i1e11406_app1.png]

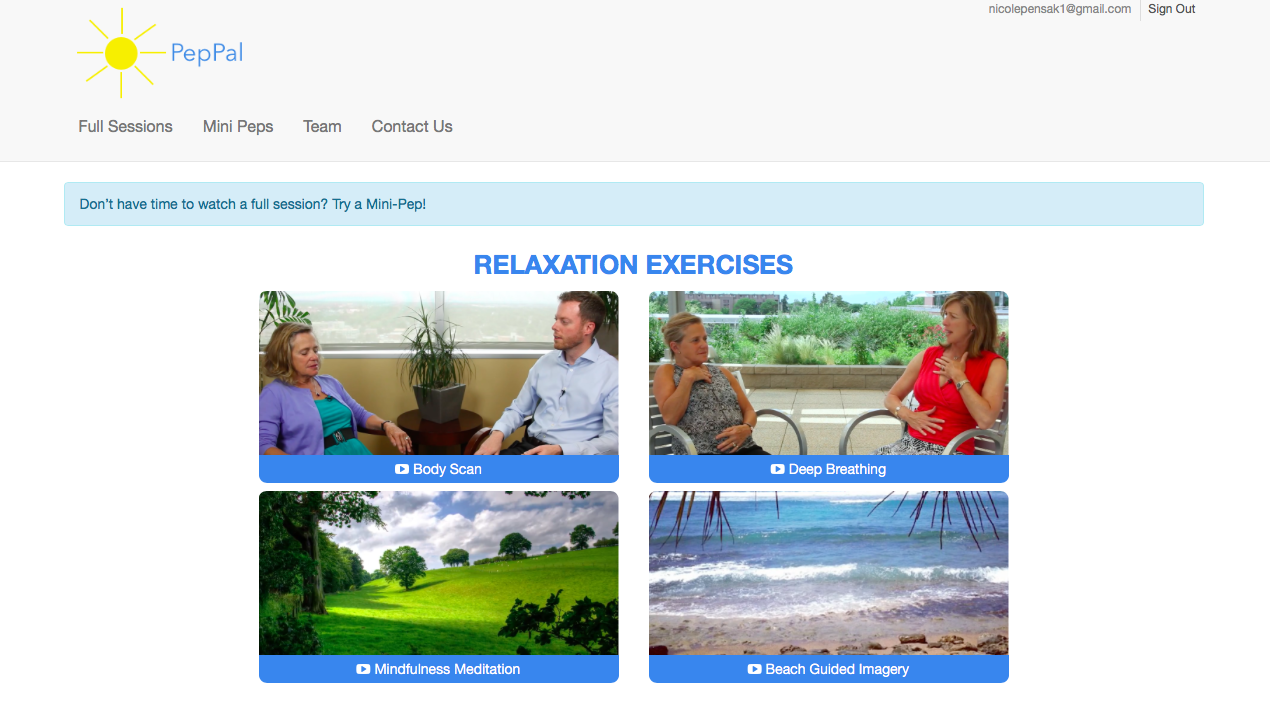

Supplement: Multimedia Appendix 2 [file cancer_v5i1e11406_app2.png]
