# Supplementary material for: Impact of a Mobilized Stress Management Program (Pep-Pal) for Caregivers of Oncology Patients: Mixed-Methods Study
Source: JMIR Cancer. 2019 May 3;5(1):e11406. doi: 10.2196/11406 (PMC6524452; doi:10.2196/11406)
Supplement: Multimedia Appendix 3 [file cancer_v5i1e11406_app3.pdf]

Qualitative Semi-Structured Interview Guide For Individual Exit Interviews

Intervention Arm

1. How were the last 12 weeks for you taking care of your loved one?

2. What have been the biggest challenges for you over the last 12 weeks while taking care of your loved one?

3. When you think about Pep-Pal, what comes to mind about your experience using it? (value judgment of it/ sense of it/ reaction to it)

4. How has Pep-Pal, the intervention, been helpful for you?
    a. In what ways in particular?
5. What was your experience like using Pep-Pal?
    a. (experience actually using it)

6. Did you watch any videos?
7. How did you watch the videos? (e.g., on laptop, computer, smartphone)?

8. Were any videos more helpful than others?
    a. Which videos and why?
    b. Tell me about those?
    c. Can you give me an example of how it was/ they were helpful?

9. Were any videos less helpful than others?
    a. Which videos and why?
    b. Tell me about those videos that were less helpful?
    c. In what way were they least helpful- can you give me an example?

10. What suggestions do you have to improve Pep-Pal?
    a. What about the sessions- would there be any thing we could do to improve the full sessions?
    b. What about the mini-peps- would there be any thing we could to improve the mini-peps?
    c. Would you want any more interactive features? For example, a chat room with other caregivers? Expert clinicians guiding discussions on different topics?
11. We are interested in adding new features to the program. Would you be interested in:
    a. Other features- direct messaging with providers?
    b. Caregiver reported patient symptom monitoring and graphs over time?
    c. Connections to local resources?
    d. Medication adherence tools?

   e. Expert clinician led forums online? – For example, an expert clinician would lead a discussion about different topics related to caregivers and self-care and provide you with the opportunity to ask questions.

12. We are interested in adding new features to the program. Would you be interested in:
    a. Would you want any other topics to be covered in the full-length modules? For example, a lot of caregivers have difficulties at work. Would you like a video for how to handle work stress or cope with work?
    b. What about a full session on how to improve sleep?
    c. What about a session on how to make meaning out of the caregiving experience?
        i. For example- to discover, reconnect, and maintain a sense of meaning even during times of stress in caregiving.
        ii. Or to- gain a greater understanding of meaning and space to explore your feelings about your loved one's illness.

13. Finally, is there anything you would like to add that we haven't covered today that is important for you and your loved one?
