# Supplementary material for: Impact of a Mobilized Stress Management Program (Pep-Pal) for Caregivers of Oncology Patients: Mixed-Methods Study
Source: JMIR Cancer. 2019 May 3;5(1):e11406. doi: 10.2196/11406 (PMC6524452; doi:10.2196/11406)
Supplement: Multimedia Appendix 4 [file cancer_v5i1e11406_app4.pdf]

Summary of caregiver-recommended future implementations for Pep-Pal (selected examples).

Exploratory Analyses	ID/ Type	Quotation
**Intimacy Session**	1026; long-term caregiver	Improving intimacy was not as helpful ... sexuality isn't an issue for us right now. He's in a lot of pain. We have intimate times of holding hands, but I can't put my arm around him.<br><br>Actual participant viewing:<br>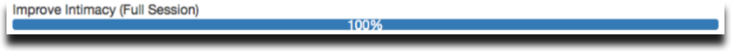
	1042; working caregiver and new caregiver	Our sexual relationship is not what it was… we are kind of recapturing it. Pep-Pal was right on, it's not all about sex. It's about intimacy. That was super good.<br><br>Actual participant viewing:<br>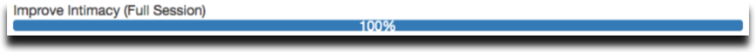
	1064; working caregiver and caregiver with a family	I liked the intimacy video ... something I hadn't seen addressed anywhere else…To be close and take time for ourselves ... not always have illness at the forefront<br><br>Actual participant viewing:<br>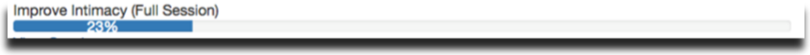
	1022; long-term caregiver and working caregiver	I remember the sex one. I didn't feel like that was very tied to, or relevant.<br><br>Actual participant viewing:<br>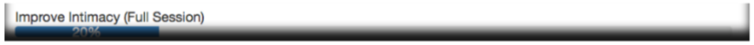
	1071; new caregiver	The sexual one … was kind of interesting. Not that I really hadn't thought about it but right now [laughter] we don't have sex.… "Oh yeah, just hugging and touching even though we aren't having sex. That's fine." That was very helpful.<br><br>Actual participant viewing:<br>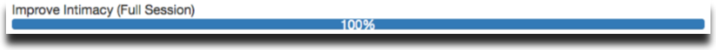

**Actress in Sessions**		
	1022; long-term caregiver and working caregiver	Just the actress, Nancy, it was just like, oh yes, I've had that same problem at home... in real life, that's not how it is. She makes it seem so simple. Like, I went out with my friends, we had a great time. I feel much better. That's not how life is.
	1042; working caregiver and new caregiver	I think her [Nancy's] father in law had more acute symptoms from chemotherapy… problems for her [Nancy] wouldn't have been the same problems I would have faced… tailoring it towards different… male versus female caregiver.
	1071; new caregiver	My favorite? I think definitely the first two or three ….maybe because those had the caregiver and brought up situations I would ponder myself. Like, "Oh. Yeah. I didn't think about that [laughter].
	1075; working caregiver	The play acting of the caregiver expressing common concerns I thought was beneficial especially, to normalize …struggles that everyone as a caregiver goes through… You feel like no one else understands. Hearing the same concerns … was … beneficial.
**Audio and mobile application**	1075; working caregiver	I would have liked some sort of **audio** option because video requires you to be watching the screen… you could listen while falling asleep or something else …driving, would be very beneficial.
	1059; working caregiver and long-term caregiver	I wish there was an **app** or an easier way to access it and it was more user friendly when it comes to **audio** and video files. A lot of times I have to myself is when I am driving and can't watch a video … if I could connect it to my radio and listen …that would be really helpful.
	1060; working caregiver and caregiver with family	I'm not tech-savvy, so I'd go to my email to access the website and type in my password get in the car …but then it'd quit. I was like, "Oh man" it wasn't user-friendly in the car. It could be an **app** that gets a better signal. That would have been helpful.
